# Inhibitory effects of carvacrol on the expression of secreted aspartyl proteinases 1–3 in fluconazole-resistant *Candida albicans* isolates

**Published:** 2016-12

**Authors:** Seyedeh Sedigheh Hosseini, Mohammad Hossein Yadegari, Masoumeh Rajabibazl, Ezzat Allah Ghaemi

**Affiliations:** 1 Department of Medical Mycology, Faculty of Medical Sciences, Tarbiat Modares University, Tehran, Iran; 2 Department of Clinical Biochemistry, Faculty of Medicine, Shahid Beheshti University of Medical Sciences, Tehran, Iran; 3 School of Advanced Technologies in Medicine, Shahid Beheshti University of Medical Sciences, Tehran, Iran; 4 Department of Microbiology, Faculty of Medicine, Golestan University of Medical Sciences, Gorgan, Iran

**Keywords:** Carvacrol, SAP1-3, *Candida albicans*, Fluconazole-resistant, *Echinophora platyloba*

## Abstract

**Background and Objectives:**

Secreted Aspartyl Proteinase (SAP) is one of the main virulence factors in the pathogenesis of *Candida.* This enzyme is encoded by a family of at least ten genes. Among these genes, the role of *SAP1-3* in mucosal infections is evident. This study aimed to investigate the expression of *SAP1-3* genes of *Candida albicans* isolates after treatment with *Echinophora platyloba* extract, carvacrol and caspofungin drug.

**Materials and Methods:**

Vaginal samples of 68 women with suspected vaginitis were obtained and cultured. *Canida albicans* species were identified using phenotypic and genotyping methods. Spectrophotometry was used to investigate the presence of *SAP* protein in the vaginal samples, and SDS-PAGE was used to confirm its protein composition. Real-time PCR was performed to ascertain the effects of subinhibitory concentrations of *Echinophora platyloba* extract, carvacrol and caspofungin on the expression of *SAP1-3* genes before and after treatment.

**Results:**

*C. albicans* was found as the abundant species (59.6%), and different amounts of *SAP* were present in all vaginal samples, which were higher than *Candida krusei* strain. The protein composition of *SAP* in *C. albicans* samples was estimated with the approximate molecular weight of 45 kDa. *mRNA* levels of total *SAP* in FLU-resistant isolates (P=0.01) were more than those of FLU-susceptible isolates (P=0.07). The findings indicated that carvacrol is effective in reduction of *SAP1-3* expression with a particular effect against FLU-resistant isolates.

**Conclusion:**

Carvacrol contains an essential oil (carvacrol); therefore, it can be considered as an alternative effective antifungal compound.

## INTRODUCTION

*Candida albicans* is an opportunistic fungal pathogen that usually lives as an infectious agent in immunocompromised patients, produces allergic reactions, and rarely leads to morbidity and mortality (1). It is also responsible for various infections, ranging from mucosal candidiasis to life-threatening disseminated candidiasis (2). Vaginal candidiasis is one of the most common and important infections in women (3), particularly during the fertile period. It is also responsible for almost 75% of all vaginal yeast infections, and other cases are mainly caused by *C. glabrata* and *C. tropicalis* (4).

Several factors are involved in the pathogenesis of *C. albicans* including adhesion, hyphae production, extracellular hydrolytic enzymes, and phenotype switching (5). Virulence of *Candida* species is directly proportional to the strength of their adhesion to different cells. *C. albicans* has the greatest adhesive ability among the *Candida* species (6).

The proteinase family is differentially regulated and expressed under a variety of laboratory growth conditions during experimental *C. albicans* infections using reconstituted human oral epithelium (RHE) (7) and *in vivo* (8). Different *SAP* genes appear to be essential for mucosal *(SAP1–SAP3)* (9) and systemic *(SAP4–SAP6)* infections. They are also involved in *C. albicans* adherence, tissue damage (10), and evasion of host immune responses (5). Therefore, inhibition of these proteinases has a protective effect for the host (11).

Fluconazole (FLC) is a member of the azole antifungal class and the most widely used drug for treatment and prevention of candidiasis. It targets essential enzymes such as ERG11 and lanosterol 14-alphademethylase in the ergosterol biosynthetic pathway (12). However, recent studies suggest that its prolonged use may contribute to development of drug resistance in *C. albicans* and other species (13).

Caspofungin is another new and effective antifungal drug that was developed as a potential antifungal and anti-pneumocystis agent (14). *In vitro*, caspofungin is fungicidal against *Candida* species (including azole-resistant species), and fungistatic against *Aspergillus* species. Caspofungin appears to have a slightly higher incidence of side effects and potential for drug-drug interactions. In addition, there is some evidence of decreased susceptibility among some strains of *Candida*, which may lessen its future utility. Comparison of caspofungin with natural drugs could pose as an interesting approach to limit the emergence and spread of these organisms, which are currently difficult to treat. Recently, there has been considerable interest in the study of plant materials as sources of new compounds for processing into therapeutic agents. One approach may be the use of extracts that have been shown to be safe potential agents in treatment of infections (15). In this context, *Echyinophora platyloba* and its major phenolic component carvacrol [2-methyl-5-(1-methylethyl) phenol] (2-isopropyl-5- methylphenol) have been given a lot of attention in recent *in vivo* and *in vitro* studies (16), and are known for their wide spectrum of antimicrobial activity. They possess multiple biological properties such as anti-inflammatory, anti-leishmanial, anti-oxidant and hepatoprotective activities (17).

Given the above, this study aimed to analyse the *in vitro* expression of *SAP1-3* genes in *C. albicans* isolates via the simple, fast and sensitive methods of spectrophotometry, phenotypic, SDS-PAGE and Real-time PCR. Additionally, changes in the expression level of *SAP1-3* virulence genes were determined by Real-time PCR after the addition of sub-inhibitory concentrations of *Echinophora platyloba* extracts, carvacrol, and caspofungin.

### Sampling, culture and identification of yeasts

This study was performed during July to December 2014 on the female patients who were referred to a qualified physician or midwife in Gorgan, Iran. Vaginal samples were obtained from 68 patients (aged 18–46 years) suffering from burning, itching, malodorou and cheesy vaginal secretions, with confirmed diagnosis by Dacron swabs. The vaginal swabs were taken from the lateral vaginal wall; for the patients presenting with infection, the swabs were taken directly from the infection areas. One of the vaginal swabs was subjected to direct examination and inoculated on the surface of YPD (yeast extract-peptone-dextrose) medium containing 2% glucose, 2% peptone, and 1% yeast extract and then incubated at 30°C for 48 hours. The purpose of each procedure was to preserve the integrity of RNAs for the subsequent analysis of *SAP* gene expression in the clinical samples. The swab samples were used to determine *Candida* colony-forming unit counts and yeast identification by CHROMagar *Candida*. In order to verify the performance of Chromagar, standard strain of *C. albicans* (ATCC10231), which produces a bright green colour, was used.

### Plant material and preparation of extract

*Echinophora Platyloba* (Iranian endemic plant) samples were collected from the mountains of Central Zagross, Chaharmahal and Bakhtiari Province during May–September, 2014. Their identity was confirmed. Harvested flowering aerial parts (leaves and flowers) were dried at room temperature for one week. The percolation method was used to obtain crude extract by stirring 100 mg of ground samples with 30 ml of pure ethanol (analytical grade; Merck, Germany) for 30 min. The samples were filtered by a Whatman no. 4 filter paper (18).

### Antifungal susceptibility test

The standard powders of FLC (F8929, Sigma-Alderich) and caspofungin (SML0425, Sigma-Alderich) were prepared in 1ml of sterile Dimethyl Sulfoxide, and carvacrol essential oil (W224502, Sigma-Alderich) was prepared in 1ml of ethanol-98%. The susceptibility of *C. albicans* strains to antifungal agents was determined by the broth micro-dilution version (19) of M27-A3 method according to the guidelines of CLSI (Clinical and Laboratory Standard Institute). After 48 hours of incubation at 30°C, MIC (minimum inhibitory concentration) was determined visually by comparing its turbidity with the drug-free growth control well. For the fluconazole, caspofungin, *Echinophora* extract and carvacrol the MIC values were defined as the lowest drug concentration for which the well was optically clear. The MIC was defined as the lowest concentration exhibiting >90% inhibition of visible growth compared to the growth of the control.

### Determination of total *SAP* by spectrophotometry and SDS-PAGE methods

*C. albicans* strain was grown in YEPD in an incubator (Heraeus) for 48 h at 27°C. The induction of *C. albicans* SAPs was performed as described previously. Briefly, 100 μl of *C. albicans* suspension was added to 10 ml of Yeast Carbon Base (YCB) (Sigma) contained BSA1%. The mixture was incubated for 7 days at 27°C in a shaker at 150 r.p.m. Thereafter, titres (c.f.u.) were determined. In brief the yeast cells were removed by centrifugation at 1500×g for 30 min. The supernatants were adjusted to pH 6.5 with NaOH to limit auto-degradation and were kept at −20°C.

A 0.1-ml volume of culture supernatant was mixed with 0.4 ml of 0.1 M citrate buffer containing BSA1% at pH 3.2 and incubated for 1h at 37°C. The reaction was stopped with 0.5 ml of 5% trichloroacetic acid (TCA) on ice for 15 min, and the mixture was centrifuged at 8–12,000 rpm for 10 min. Then the absorbance was read at 280 nm against distilled water, which was blank (20). Standard strain of *C. albicans* (ATCC10231) was used as the positive control. SDS-PAGE was done according to the method of Lammeli (21).

### RNA extraction & analysis of *SAP* gene expression

In order to analyze the expression of *SAP1-3* genes, RNA was extracted from *C. albicans* isolates before and after treatment with the MIC concentration of each extract using Trizol reagent (Invitrogen Co., 15596026) according to the manufacturer’s protocol. Complementary DNA was synthesized using revert aid first strand cDNA synthesis kit followed by DNaseI (Thermo Fisher Scientific Co., EN0521) treatment.

The cDNA was synthetized using the iScript cDNA synthesis kit (Fermentase Co., k1622) according to the manufacturer’s protocol. ACT1 primers for Real-time PCR analysis were designed using the PRIMER3 web-based software (http://frodo.wi.mit.edu/primer3). The primers were checked for specificity through the BLAST search available on the NCBI website (http://blast.ncbi.nlm.nih.gov/Blast.cgi ). A primer set for the hemochromatosis gene was designed to be used as an internal control (Table 1).

**Table 1.** Primers used for Real time-PCR analysis of the *SAP* and control genes’ expression

**Accession number**	**Sequence(5′–3′)**	**Primer**	**Product size (bp)**
XM-712960.1	GTTGGTTTTGGTGGTGCTTC	SAP1F	200
	GCAGCATTGGGAGAGTTGAG	SAP1R	
XM-705969.1	TGTGGATTCAGGTACCACCA	SAP2F	192
	GCAAATTCGGAAGCTGGA	SAP2R	
XM-718117.1	TGGTCAAGGACAAGATCCAA	SAP3F	231
	CCAATCCCTAAAATCCCTTG	SAP3R	
XM-717232.1	CCAGCTTTCTACGTTTCC	ACT1R	209
	CTGTAACCACGTTCAGAC	ACT1F	

### Real-time PCR

The RealQ Plus 2x Master Mix Green (Ampliqon) was used to determine the relative level of *SAP1–3* mRNA transcripts with actin 1 (ACT1) as a reference housekeeping gene. The PCR process was performed according to an optimized protocol. The relative quantification of *SAP1–3* gene expression was performed by Syber green dye. Each test was performed in triplicate, and mean values of the relative expression were determined for each *SAP* gene.

The expression levels of *SAP1-3* were evaluated using the 2^−ΔΔCt^ method; where, C_t_ is the average threshold of cycles from three independent experiments (7). Data were presented as the fold change in gene expression normalized to the *18SrRNA* gene as a control.

### Statistical analysis

The obtained data were analysed using SPSS-18 statistical software and the Kruskal-Wallis test in groups. Mann-Whitney’s U-test was done to analyse the inter-group relationships. All analyses were performed at 95% confidence level. The REST (Relative Expression Software Tools) software (2009) was also employed to calculate the ratio between the amount of target molecule and reference molecule within the same sample. In this model, the target gene expression was normalized by *ACT1* expression, which is a non-regulated reference gene. The normalized value was then applied to compare differential gene expression in different samples.

## RESULTS

42 out of the 68 direct smeared samples were culture positive with 5 different yeast strains; 47.8% of which were *C. albicans*. Other species including *C. parapsilosis* (24%), *C. glabrata* (17.8%), *C. tropicalis* (8.4%) and *C. krusei* (2%) were detected by the phenotypic methods and RFLP-PCR (data not shown).

### Determination of total *SAP* by spectrophotometry

The activation of *SAP* enzymes based on the amount of substrate BSA decomposition was investigated by spectrophotometry and light absorption at 280 (8).

In the test tube containing *SAP* enzyme and the substrate, these enzymes caused to break down of BSA proteins, and small peptides were achieved. In the control tube, standard strain of *C. albicans* was considered as a strains that has *SAP* enzyme and *Candida krusei* was considered as low value of *SAP* enzyme level; so with the addition of TCA to the samples, substrate and enzyme precipitated and only small peptides derived from proteolysis of the enzyme can be studied by spectrophotometer. The results showed that the enzyme had high proteinase activity in comparison with the negative control, and this case with a positive control strain of standard strains of *C. albicans* was confirmed (Table 2).

**Table 2.** Determination of *SAP* activity by spectrophotometry in *C. albicans* isolates

**Strain**	**Mean SAP level** (μg/ml)
C. albicans* (ATCC10231)	6.82±1.60
FLU-resistant (n=7)	9.05±1.10
FLU-susceptible(n=13)	2.82±1.68
C. Krusei* (ATCC6285)	0.01±0.08

*SAP*’s average level in the four tested groups: Significant differences within the groups were determined by Kruskal-Wallis test (*n* = 20, *P* < 0.05). Significance of differences between the groups was assessed by Mann-Whitney’s *U*-test, (*n* = 20, *P* < 0.05).

* ATCC 10231 *C. albicans*=Positive control

* ATCC6285 *C. krusei*=Negative control

### Confirmation of total *SAP* by SDS-PAGE

**Fig. 1.**
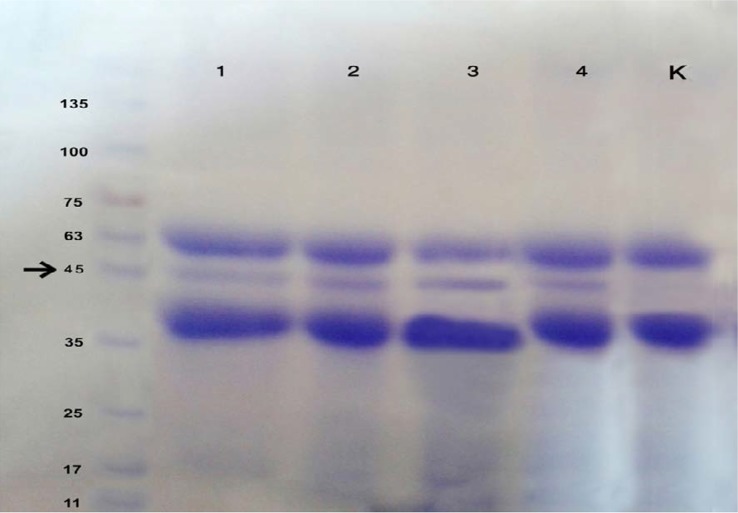
*SAP* protein isolates of *C. albicans* in the BSA medium. 1. Standard strain of *C. albicans*, 2–4. *C. albicans* isolates, K-Standard strain of *C. krusei* stained with coommassie blue.

Comparison of the standard strain as positive control with the isolates was done by the SDS-PAGE method. In this study, protein markers with wide range of molecular weight from 14.4 to 116 kDa was used. The results showed one protein band in the *SAP* extract with a molecular weight of almost 45KD (Fig. 1).

The SDS-PAGE results demonstrated that the *SAP* level of the 35% isolates increased compared with the standard strain *C. albicans* as a positive control. The rest of *C. albicans* isolates showed significantly different *SAP* expression compared with the standard strain *C. kruzei* as a negative control (P<0.05).

### *In vitro* susceptibility analysis

In order to assess the antifungal effect of the *Echinophora*, carvacrol, and caspofungin, *C. albicans* isolates were exposed to different concentrations of each extract and drug. The lowest MIC of the clinical isolates of *C. albicans* to the extract of *Echinophora platyloba* and carvacrol was 64mg/ml and 0.25 μg, respectively.

Evaluation of the MIC values showed that carvacrol was active against all the tested strains. The highest level of activity was observed against 13 FLU-susceptible isolates with a MIC value of 0.25–1.23 μg/ml (Table 2).

The MIC values of *Echinophora* ethanolic extract ranged from 64mg/ml to 512 mg/ml against the FLU-resistant isolates. Other *Echinophora* MIC values ranged from 1mg/ml to 64mg/ml against the FLU-susceptible *C. albicans* isolates. Carvacrol was found as the active constituent of *Echinophora*, with MIC values ranging from 0.03 to 23μg/ml (Table 2). Both *Echinophora* and carvacrol showed a broad spectrum of activity against a variety of pathogenic yeasts including fungi with decreased susceptibility to fluconazole (Table 3). Nevertheless, carvacrol proved to be more active against *candida* isolates, as did *Echinophora platyloba.*

**Table 3.** *In-vitro* susceptibility testing of Fluconazole, Caspofungin, *Echinophora platyloba* and Carvacrol against clinical isolates of *C. albicans* (n = 20) by microbroth dilution assay (μg/ml) (CLSI method)

**Species (No. of isolates)**	**Antifungal agent**	**Range**	**50%**	**90%**	**Number of resistance (%)**
	Fluconazole	0.031–128	8	16	7(35%)
*C. albicans* (20)	Echinophora	31–512000	16000	32000	9(45%)
	Carvacrol	0.03–8	1.23	2.46	3(15%)
	Caspofungin	0.03–12	1	2.3	6(30%)

Resistance is defined as the following MIC in Fluconazole ≥ 64; *Echinophora* > 32000; Carvacrol ≥ 3.0 and Caspofungin ≥ 2.0.

### *SAP* 1–3 expression in *C. albicans* isolates

*SAP1–SAP3* expression by *C. albicans* in both FLU-susceptible and FLU-resistant isolates are shown in Table 3. FLU-susceptible *C. albicans* isolates showed lower expression for the target genes while FLU-resistant isolates had a significant increase in *SAP1-3* gene expression. Higher *SAP1-3* expressions were found in strains 14–20 (FLU-resistant) compared with strains 1–13 (FLU-susceptible). The mean of the targeted gene’s expression level between the resistant and susceptible strains was significantly different (P <0.008) (Table 4).

**Table 4.** *SAP1-3* genes expression in *C. albicans* isolates

Subject group	Strain number	Expression of gene (%)		

		SAP1	SAP2	SAP3
FLU-susceptible isolate	13	90	89	53
FLU-resistant isolate	7	89	79	68
*C. albicans* ATCC10231	Positive control	100	82	51

**NOTE**. Data are no of subjects (%) who were positive for the expression of a particular gene. Detection of *SAP1-3* mRNA expression in the susceptible and resistant *C. albicans* isolates. The total percentage of subjects expressing each *SAP* gene is illustrated in each group: Susceptible (n=13) and Resistant (n=7). Data are shown as means ± SD from at least three experiments.

### Effect of *Echinophora Platyloba*, carvacrol, and caspofungin drug on expression of *SAP1–3.*

**Fig. 2.**
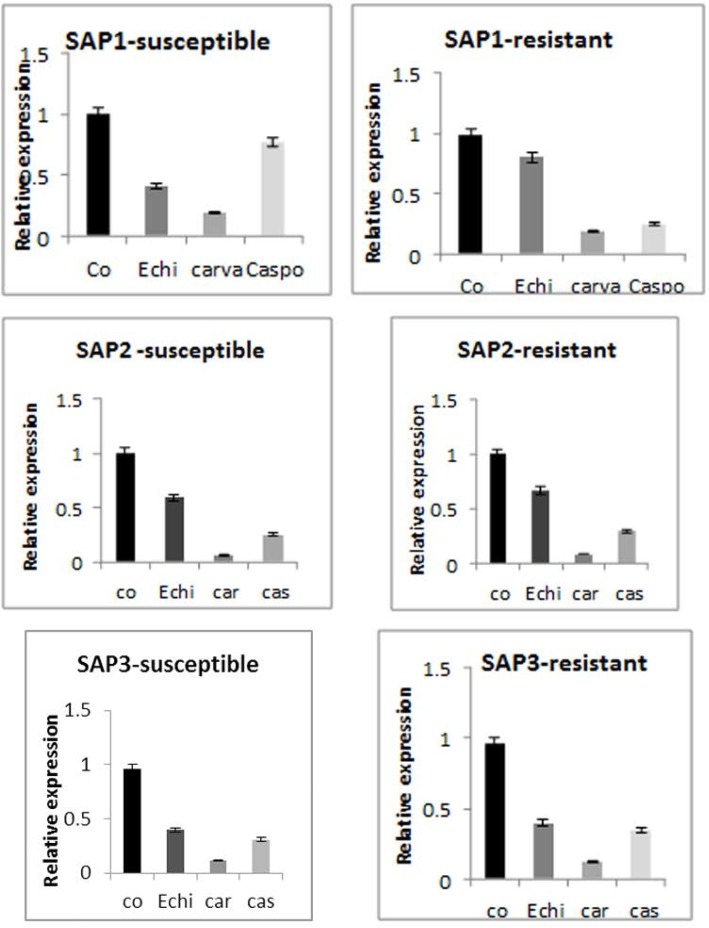
Real time PCR for each gene was performed using 1 μg of total RNA. Gene expression is indicated as a fold-increase relative to control (black bars), carvacrol (gray bars), *Echinophora* (dark gray bars) and caspofungin (light gray bars). The results were normalized against ACT1 housekeeping gene expression, which was assigned a value of 1(P<0.05).

*C. albicans* isolates (susceptible and resistant) were tested extensively for *SAP* activity in the presence and absence of sub-MIC concentrations of *Echinophora platyloba*, carvacrol, caspofungin, and fluconazole. Expression of *SAP1* in susceptible and resistant isolates decreased after treatment with *Echinophora* by about 3.6 and 2 fold, respectively (P<0.02) (Fig. 2(a, d)). Expression of *SAP1* significantly decreased after treatment with carvacrol inhibitors in both isolates (P<0.001) (Fig. 2(a, d)). Expression of *SAP1* in susceptible and resistant strains decreased by about 4.7 and 4 fold, respectively after treatment with caspofungin (P<0.01) (Fig. 2(a, d)). Expression of *SAP2* in the resistant and susceptible isolates decreased by about 2 and 1.6-fold after treatment with *Echinophora* (P<0.3) (Fig. 2(b, e)). Expression of *SAP2* significantly decreased following treatment with carvacrol inhibitors in both strains relative to the control (p<0.034) (Fig. 2(b, e)). Expression of *SAP3* in susceptible and resistant isolates decreased by about 3.2 and 2.8 fold, respectively after treatment with caspofungin (P<0.3) (Fig. 2(c, f)).

Expression of *SAP1-3* was significantly altered when *C. albicans* isolates were challenged with sub-MIC concentration of different antifungal agents (Fig. 2; P<0.05). Carvacrol down-regulate *SAP1-3* expression more than *Echynophora* and caspofungin, and the difference was statistically significant (P<0.03). Of all the agents tested, carvacrol caused the highest down-regulation of *SAP1-3* expression (Fig. 2).

## DISCUSSION

Incidence of *Candida* species has shown an increasing trend in recent years, mostly due to rising number of immunosuppressed patients and widespread use of broad-spectrum antibiotics that leads to the increase of drug-resistant *Candida* strains (1).

This study aimed to determine the *in vitro* activity of *SAP1-3* in several isolates of *C. albicans* from vaginal samples, cultured in the YEP medium and ascertain the effects of subinhibitory concentrations of *Echinophora platyloba* extract, carvacrol and caspofungin on the expression of secreted aspartyl proteinases 1–3 before and after treatment. All the tested isolates (20) were found positive for production of *SAP* (Table 2). The results showed that 40 out of 68 patients were infected by yeast, 47.8% of which had *C. albicans*. After *SAP* confirmation in the vaginal samples by spectrophotometry (22), the protein composition of *SAP* was analysed by SDS-PAGE, and a band with the weight of almost 45 kDa (Fig. 1) (23) was observed, which corresponds to the study of Zaugg and et al (24). The average level of *C. albicans SAP* was significantly more in the FLU-resistant isolate than in the FlU-susceptible isolate.

The study findings also showed increased gene expression of *SAP1-3* in FLU-resistant *C. albicans* isolates compared to the susceptible isolates. This indicates a clear link between the expression of *SAP 1–3* exposure to antifungal agents and drug resistance.

The results of the present study imply that the majority of *SAP*s important for pathogenicity during vaginal infections with *C. albicans* are *SAP1–3*. We found that the expression of *SAP1* gene of *C. albicans* isolates was more than that of *SAP2* and *SAP3* (Table 4). The observation of high-density *SAP1* and *SAP3* increased further in FLU-resistant *C. albicans* isolates (Table 4) (25). However, the expression levels of *SAP1* and *SAP3* were significantly greater in the FLU-resistant isolates than FLU-susceptible isolates. Therefore, they play a major role in adhesion, and colonization mucosal surface conferred drug resistance of *C. albicans* isolates (26). Also we found that the expression level of *SAP2* was higher in the FLU-susceptible isolates than in FLU-resistant isolates; this shows the role of *SAP2* in the development of *C.albicans* pathogenicity (Table 3) (27–28). Therefore, it seems necessary to find new drug agents having the least toxicity, and side effects in the treatment of *Candida* infections.

*Echinophora platyloba*, carvacrol, FLU and caspofungin are used to inhibit *SAP* expression with wide-spectrum of antifungal activities. Evaluation of MIC values showed that *Echinophora* was active against all the tested strains. The highest level of activity was observed against FLU-resistant isolates, with a MIC value of 64mg/ml (Table 3).

Essential oils and their components have been timely honored for their pharmaceutical properties (30). Carvacrol containing essential oil has been reported to have antiseptic, antibacterial, antiviral and antifungal activities (16).

*Echinophoral* and carvacrol showed a broad spectrum of activity against a variety of pathogenic yeasts, including fungi with decreased susceptibility to FLU (Table 3). Carvacrol has been proved to be more active against *Candida* strains, and the fungistatic and fungicidal properties of essential oil probably associated with its high carvacrol content (29–31).Thirteen of 20 isolates of *C. albicans* grown under *SAP*-inducing conditions responded in a dose-dependent manner to partial growth inhibition by caspofungin and FLU by decreasing their extracellular *SAP* activity. This finding shows that carvacrol downregulates the gene expression of *SAP2* activity in Flu-susceptible and Flu-resistant isolates comparing to Echinophora and caspofungin (Fig. 2) (32). Also downregulation of *SAP1* and *SAP3* expression was observed in both FLU-resistant and susceptible isolates. Therefore, carvacrol is effective in reduction of *SAP1–3* expression, with a particular effect against FLU-resistant isolates. This confirms a general relationship between FLU-susceptibility and relative expression and activity of *SAP.*

Considering the above findings, particularly the possible mechanisms of action, which can induce side effects in humans, these antifungals need further investigations. Toxicity studies, improved formulations, determination of optimal concentrations for clinical applications and comparative studies on the therapeutic efficacy of carvacrol with drugs currently in use are recommended to further control of fungal infections.
